# Prevalence and Predictors of Antibiotic Prescriptions at Primary Healthcare Facilities in the Dodoma Region, Central Tanzania: A Retrospective, Cross-Sectional Study

**DOI:** 10.3390/antibiotics11081035

**Published:** 2022-07-31

**Authors:** Richard James Mabilika, Gabriel Shirima, Emmanuel Mpolya

**Affiliations:** 1 Department of Global Health and Biomedical Sciences, School of Life Sciences and Bioengineering, The Nelson Mandela African Institution of Science and Technology, Arusha P.O. Box 447, Tanzania; gabriel.shirima@nm-aist.ac.tz (G.S.); emmanuelmploya@nm-aist.ac.tz (E.M.); 2 Department of Biomedical Sciences, School of Medicine and Dentistry, The University of Dodoma, Dodoma P.O. Box 395, Tanzania

**Keywords:** antibiotic, resistance, prescription, bacteria, diagnosis, prescriber

## Abstract

Background: Accelerated by the misuse or overuse of antibiotics, antibiotic resistance remains a global public health threat. We report the prevalence and predictors of antibiotic prescriptions in primary healthcare facilities in Dodoma, Tanzania. Methods: This retrospective cross-sectional study by medical records review was conducted in the Dodoma region, Central Tanzania. Results: In this study, children < 5 years accounted for over 45% (474/1021) of the patients consulted. The majority, 76.3% (779/1021), of consultations had an antibiotic prescribed; amoxicillin and cotrimoxazole were the most prescribed. Over 98% (766/779) of the antibiotics prescribed were on the National Essential Medicines List, but only 45% (429/779) of the antibiotic prescriptions adhered to the Standard Treatment Guidelines. The prescribing of antibiotics by clinical officers was almost 2.55 times higher than that among medical doctors (Odds Ratio (OR) = 2.546; 95% Confidence Interval (CI): 1.359, 4.769; *p* = 0.0035). Patients with pneumonia and upper respiratory tract infection were 15.9 (OR = 15.928; 95% CI: 2.151, 17.973; *p* = 0.0067) and 2 (OR = 2.064; 95% CI: 1.184, 3.600; *p* = 0.0106) times more likely to be prescribed antibiotics, respectively. Conclusions: We, therefore, report high rates of antibiotic prescriptions, poor adherence to standard treatment guidelines and high levels of antibiotic prescribing practices among prescribers with a diploma in clinical medicine.

## 1. Introduction

Antibiotic resistance is one of the greatest public health issues threatening the prognosis of bacterial infections globally [1]. It develops naturally whenever bacteria come in contact with antibiotics and is much more accelerated by the misuse or overuse of antibiotics in the human and animal health sectors [2]. Antibiotic resistance increases the risk of common bacterial infections no longer responding to antibiotics that are used to inhibit them, resulting in prolonged morbidity and increased mortality [3].

In low-income countries, problems such as poor sanitation and waste disposal control programs, lack of clean and safe water, unregulated community prescribing and sale of antibiotics and an ever-increasing human population are among the key factors fueling the spread of antibiotic-resistant pathogens [1]. In 2019 Sub-Saharan Africa accounted for the highest death rate attributed to antibiotic resistance (99 deaths per 100,000), followed by South Asia (77 deaths per 100,000) [4]. The global annual death toll from antimicrobial resistance (AMR) is now estimated to be approximately 700,000 and will amount to 10 million in 2050, forcing another 24 million people into extreme poverty [5]; this is much more the case in low-income countries [6].

The use of antibiotics in clinical practice is mainly manifested by both the transactions of these agents in community drug shops and the antibiotic prescribing practices by healthcare workers across the entire healthcare spectrum [2,7,8]. In Tanzania, the health system follows a referral hierarchy where dispensaries and health centres form the primary healthcare block serving a population of 5000 and 50,000 at the village and ward levels, respectively [9]. Higher-level healthcare block constitutes district hospitals, regional referral hospitals and zonal referral hospitals in that order [9]. The primary healthcare-prescribing work-force ranges from prescribers with a certificate in clinical medicine (two years of medical college training), commonly referred to as medical assistants, to prescribers with a diploma in clinical medicine (three years of medical college training), commonly referred to as clinical officers to medical doctors (five years university degree in medicine) [9].

Antibiotic-prescribing practices in primary healthcare facilities in Tanzania largely rely on clinical examination and patients’ medical history rather than microbiological evaluation [10]; this increases the potential for their misuse and/or overuse. A study involving patients in primary healthcare facilities conducted in Dar es salaam, Kilimanjaro, Mwanza and Mbeya Tanzania reported over 65% of antibiotic prescriptions [11]. In another study on antibiotic prescriptions among health-insured patients in Dar es Salaam, 46.4% of all the patients were prescribed antibiotics [12]. Antibiotic prescriptions in both studies were higher than the optimal (<30%) as per the index of rational drug prescribing (IRDP) [12].

The Tanzanian National Action Plan on AMR (NAP–AMR) was launched in 2017 with the aim of reducing the burden of AMR in the country and contributing to the AMR global data [13]. Among the achievements made by NAP–AMR include the establishment of a multisectoral coordinating committee on AMR activities, establishment of the human and animal surveillance sites, creation of community-based AMR stewardship awareness campaigns and the availability of AMR stewardship guidelines at health facility levels [14]. Nevertheless, accountability, reporting and feedback mechanisms, transparency and sustainability of the AMR plans remain a challenge [14].

The prescribing of antibiotics by healthcare practitioners across the entire healthcare referral hierarchy has to abide by the Standard Treatment Guidelines (STG) and the National Essential Medicines List (NEMLIT), prepared and updated every three years by the Ministry of Health [9]. While many studies have reported on antibiotic prescribing practices and the resulting resistance profiles in secondary and tertiary healthcare facilities, those of primary healthcare settings remain underrepresented [15]. In this study, we report on the prevalence and predictors of antibiotic prescriptions in primary healthcare facilities in Dodoma region, Central Tanzania.

## 2. Material and Methods

### 2.1. Study Design, Sites and Settings

This was a retrospective cross-sectional study by medical records review conducted in two districts of the Dodoma region in Tanzania. The two districts were randomly selected from a group of rural districts and urban districts of the Dodoma region to represent the rural and urban settings, respectively. Dodoma City Council houses the central business district of Dodoma City and is an urban setting. Chemba District Council represents the rural setting. Dodoma City Council (urban) has a population size of approximately 460,000, and Chemba District Council (rural) has a population size of approximately 250,000 [16]. Dodoma City Council covers approximately 2769 square kilometres and has 4 hospitals and 61 primary healthcare facilities; Chemba District Council covers a total of 7653 square kilometres with 39 primary healthcare facilities [16].

### 2.2. Data Sources and Study Population

The sampling frame of this study was primary healthcare facilities in the two districts. Data collection was based on the WHO indicators of antibiotic prescribing [17] and the Tanzanian Standard Treatment Guidelines [18]. One-year retrospective prescribing data from January 2020 to December 2020 were randomly sought and recorded from the selected health facilities. Medical records were used to gather retrospective information pertaining to patients’ age and sex, qualifications of prescribers, empirical diagnosis and the type and the number of medicines prescribed.

### 2.3. Sample Size Calculation

The sample size calculation was calculated based on the unknown prevalence (*p*) of 50% with 95% confidence, and the degree of precision employed was 5%, with a design effect of 1 and a response rate of 90% [19]. This gave us a minimum sample size of 427 prescriptions/medical records. Given that this was a retrospective medical records review, this sample size provided assurance of having more than 10 subjects per variable, which is considered ideal for performing regression analyses [20,21].

### 2.4. Sampling Technique

The sampling technique employed was multistage stratified random sampling method. The Dodoma region is divided into 7 districts [16], which, depending on their population densities, the presence of modern infrastructure of roads and railways and a high density of shops—reflective of higher household income—were divided into rural (Chemba, Bahi, Mpwapwa, and Chamwino districts) and urban (Dodoma City Council, Kondoa and Kongwa) categories. One district was then randomly selected from each of the two groups to obtain an urban and rural representative district. The two districts randomly selected were Chemba District Council from the rural category and Dodoma City Council from the urban category. In the second stage, probability proportional to size sampling was used in deciding the total number of prescribing consultations to be included in the study from the two localities. In 2016, there were 93,339 households in Dodoma City Council and approximately 47,100 households in Chemba District Council [16]. Proportionately, we would require twice the number of prescribing consultations in Dodoma City Council as in Chemba District Council. A total of 631 and 390 consultations were recorded from 21 and 13 primary healthcare facilities in Dodoma City Council and Chemba District Council, respectively.

### 2.5. Data Collection

Data were collected using an Open Data Kit (ODK) digital questionnaire. The questionnaire was adopted from a similar study [22] and modified to incorporate patients’ social demographics, diseases diagnosed, adherence to the Tanzanian standard treatment guidelines, prescribers’ qualifications, types and number of drugs prescribed and the rural and urban variables. The modified English questionnaire was translated into Swahili for easy comprehension among data collectors, and its suitability was tested in a pilot study before it was adopted for use in this study. Data collectors were recruited and oriented to the digital data collection questionnaires using supplied Android smartphones. Ethical clearance was sought from and granted by the Northern Zone Health Research Ethics Committee (Approval code: KNCHREC0020). Authorities in Dodoma were consulted for the relevant permissions for the study, and consent was obtained from heads of respective healthcare facilities by signing the NM-AIST consent form.

### 2.6. Inclusion and Exclusion Criteria

All records from prescription books in primary healthcare facilities recorded from January 2020 to December 2020 were deemed relevant; however, records from maternity clinics were excluded.

### 2.7. Data Analysis

Antibiotic prescription was an outcome variable in this study, and other independent variables included diagnosis as well as social and demographic characteristics. SAS version 9.4 was used for data analysis, and the significance of all statistical tests was set at the 5% level of significance. Basic descriptive statistics, such as frequency and percentages, were evaluated and used to describe baseline characteristics. A chi-square test of association was also employed to test the association of diseases diagnosed and the status of antibiotic prescription.

The outcome variable had two responses (Yes/No); thus, a binary logistic regression model was used to determine factors associated with prescribing antibiotics.

The results of the model are presented in the form of regression parameter estimates and estimated odds ratios (ORs). The estimated ORs, determined by taking the exponent of the regression parameter estimates, show the increase or decrease in the likelihood of the outcome at a given level of the independent variable compared to those in the reference category. The estimate of OR > 1 indicates that the likelihood of receiving antibiotics at a given level of the independent variable is greater than that of the reference category. Similarly, the estimate of OR < 1 specifies that the chance of receiving antibiotics at a given level of the independent variable is less than that for the reference category.

## 3. Results

### 3.1. Patient and Prescriber Characteristics

Of the 1021 retrospective prescribing consultations in this study, 61.8% (631/1021) were from Dodoma City Council. The majority (94.12%; 961/1021) of the consultations were those recorded from public primary healthcare facilities, and 5.88% accounted for consultations recorded in private and other faith-based primary healthcare facilities. Children under the age of five accounted for over 45% (474/1021) of all the consultations. There were more consultations involving females (54.55%; 557/1021) than those involving males. Medical doctors (holders of a degree in medicine, acquired after 5 years of study), Clinical officers (holders of a diploma in clinical medicine, acquired after 3 years of study), and medical assistants (holders of a certificate in clinical medicine, acquired after 2 years of study) were the prescribers recorded in this current study. The majority (94.61%; 966/1021) of the antibiotic prescriptions were made by prescribers with a diploma in clinical medicine (Table 1).

**Figure 1:**
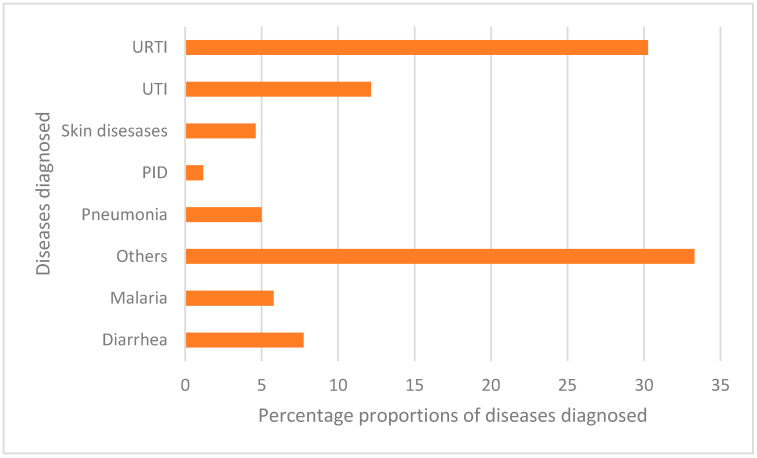
Proportions of individuals diagnosed with common diseases (Others: A group of conditions not presented in the chart).

The most common empirical diagnoses were upper respiratory tract infections (URTIs), urinary tract infections (UTIs) and diarrhoea, which accounted for 30.3%, 12.1% and 7.7%, respectively (Figure 1).

**Figure 2:**
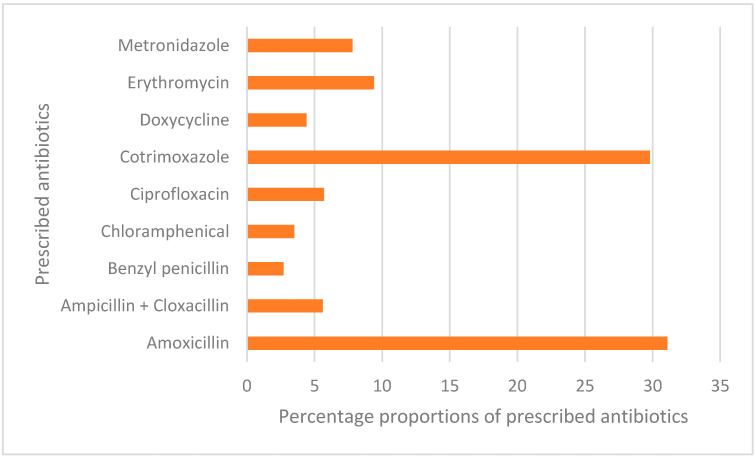
Commonly prescribed antibiotics.

Of the 1021 prescribing consultations recorded in this study, 76.3% (779/1021) had an antibiotic prescribed, with amoxicillin and cotrimoxazole accounting for over 60% of all the prescribed antibiotics (Figure 2).

The majority (98.3%; 766/779) of the antibiotics prescribed were listed on the National Essential Medicines List (NEMLIT) [12] and were all in the ‘*access*’ category as per the WHO *AWaRe* (Access, Watch and Reserve) antibiotic classification system as classified in the Tanzanian Standard Treatment Guidelines (STG) [12]. About 45% (350/779) of the antibiotic prescriptions were not aligned to the empirical diagnoses as per the Standard Treatment Guidelines (STG).

### 3.2. Stratification of Social, Demographic and Clinical Predictors for Antibiotic Prescription Status

The proportions of antibiotic prescriptions among male patients (78.8%) were significantly higher (*p* value = 0.0382) than that among female patients (73.3%). The most common diagnoses for which antibiotics were prescribed included urinary tract infection (UTI) (100%; *p* value < 0.0001), upper respiratory infection (82.1%; *p* value = 0.05), pneumonia (98.04%; *p* value = 0.0002), diarrhea (73.4%; *p* value = 0.53), skin diseases (53.2%; *p* value = 0.0001) and pelvic inflammatory diseases (PIDs) (41.7%; *p* value = 0.101) (Table 2). Nevertheless, a significant proportion (54.2%; *p* value = 0.0001) of patients diagnosed with malaria were prescribed antibiotics.

### 3.3. Binary Logistic Regression Analysis of Antibiotic Prescriptions by Social, Demographic and Clinical Predictors

#### 3.3.1. Univariate Logistic Regression Analysis

Female patients were 27.4% less likely to be prescribed antibiotics (crude OR = 0.726; 95% CI: 0.552, 0.984; *p* = 0.0386). Compared to medical doctors (degree in medicine), there was an almost twofold increase (crude OR = 1.989; 95% CI: 1.106, 3.577; *p* = 0.0217) in the odds of antibiotic prescriptions among clinical officers (diploma in clinical medicine). Patients diagnosed with upper respiratory tract infection and pneumonia were 1.913 times (crude OR = 1.913; 95% CI: 1.354, 2.701; *p* = 0.0002) and 16.678 times (crude OR = 16.678; 95% CI: 2.271, 120.270; *p* = 0.0055) more likely to be prescribed antibiotics, respectively, than patients who did not have these conditions. Patients with malaria, skin diseases and other disease conditions were 66% (crude OR = 0.340; 95% CI: 0.200, 0.583; *p* > 0.0001), 65.9% (crude OR = 0.341; 95% CI: 0.193, 0.600; *p* > 0.0003) and 31.4% (crude OR = 0.686; 95% CI: 0.511, 0.908; *p* > 0.0112) less likely to be prescribed antibiotics, respectively (Table 3).

#### 3.3.2. Multivariate Logistic Regression Analysis

In the adjusted model, antibiotic prescriptions among prescribers with a diploma in clinical medicine were almost three times higher than those reported among those with a degree in medicine (adjusted OR = 2.511; 95% CI: 1.343, 4.692; *p* = 0.0039). Patients with pneumonia and upper respiratory tract infection were 16 (adjusted OR = 15.918; 95% CI: 2.151, 17.973; *p* = 0.0067) and 1.709 (adjusted OR = 1.709; 95% CI: 1.129, 2.587; *p* = 0.0113) times more likely to be prescribed antibiotics, respectively. Patients with malaria and skin diseases were 70.7% (adjusted OR = 0.293; 95% CI: 0.160, 0.504; *p* = 0.0001) and 67.9% (adjusted OR = 0.321; 95% CI: 0.184, 0.631; *p* = 0.0008) less likely to be prescribed antibiotics compared those with other diseases (Table 3).

## 4. Discussion

In this study, the majority of the consultations in both the rural (74.1%; 289/390) and urban (77.7%; 490/631) districts had an antibiotic prescribed. The observation in our current study is higher than that reported in Cameroon, where 36.7% of the consultations recorded in primary healthcare were prescribed antibiotics [23]. Additionally, a study on antibiotic prescriptions in public primary healthcare facilities in Ethiopia reported over 56% of antibiotic prescriptions [22]. In a review on antibiotic prescriptions in primary healthcare facilities involving 48 studies from 27 low- and middle-income countries, the prevalence proportion of antibiotic prescriptions was also reported at 56% [24]. The findings of this current study show alarmingly high levels of antibiotic prescriptions, an observation likely to be influenced by poor adherence to standard treatment guidelines among primary healthcare prescribers [25] and the absence of laboratory facilities in these lower-level healthcare facilities [25].

This study reports low adherence (55%; 429/779) to standard treatment guidelines (STG) among primary healthcare prescribers. More than half (54.2%; 32/59) of the malaria cases in this study were prescribed antibiotics (cotrimoxazole and amoxicillin) against STG. Additionally, even though the Tanzanian STG recommends that PID be treated with a combination of antibiotics, 60% of the PID cases in this study were not prescribed antibiotics. This is supported by the previous study in primary healthcare facilities in Dodoma, Tanzania, where 25% of malaria cases were also prescribed antibiotics against STG [25]. This finding was further supported by a study conducted in Cameroon, where uncomplicated malaria was treated with antibiotics against treatment guidelines [14]. Administering antibiotics to patients whose disease is not bacterial is a gross misuse of these vital medicines and paves the way for the development of antibiotic resistance [2]. The high levels of non-adherence to standard treatment guidelines by prescribers in primary healthcare facilities could be attributed to stock-outs of essential medicines, poor emphasis on the use of STGs and/or the overt absence of the STG books in these lower cadre settings.

In this current study, the odds of antibiotic prescriptions by the clinical officers (diploma in clinical medicine) were almost three times higher than those of medical doctors (Medical Doctor degree). In a study on the use of antibiotics for cough and/or diarrhoea in northern Tanzania, clinical officers were also among the prescribers associated with excessive and inappropriate antibiotic prescriptions for nausea, vomiting and diarrheal conditions [26]. Clinical officers were also most likely to prescribe wrong dosages to patients (doses that were too high or too low) [26]. Studies by [27] on the prescribing practices in Dar es Salaam Tanzania have reported, among others, costs and availability of drugs as determinants of prescribing decisions; rather than positive microbiological results. Coughs, colds and diarrhoea reported in community drug outlets and primary healthcare facilities are mostly prescribed and dispensed with antibiotics [28]. Clinical officers constitute a majority of prescribers across the primary healthcare spectrum in Tanzania [9]; they are the first-contact healthcare personnel and thus a key AMR stewardship intervention point [9]. There is thus a need for regular antibiotic stewardship campaigns and training on the importance of rationality in antibiotic prescription and use among prescribers in primary healthcare as one key strategy in curbing antibiotic resistance.

Among the medical conditions recorded, symptomatic pneumonia was an important predictor for antibiotic prescriptions in this study. Similar findings have been reported in Uganda [29], where the majority of empirically diagnosed pneumonia cases received antibiotics. Confounded by delayed laboratory diagnosis, the prognosis for pneumonia is especially poor in children, justifying the urgency for empirical treatment [30]. Rapid and reliable diagnostics are thus key to rational antibiotic prescriptions.

Over half (53.2%; 25/57) of patients with dermatological conditions in this study were prescribed systemic antibiotics, being a 66.9% decrease in the odds of systemic antibiotic prescriptions as compared to patients with other disease conditions. The observation in this current study is lower than that reported in a study elsewhere on the prescription of antibiotics among dermatology patients, where the odds of empiric antibiotic prescriptions were almost five times higher compared to all others [31]. In another study involving 683 patients in Australia, 35% (239/683) of the cases were prescribed topical antibiotics, 44.8% (306/683) had systemic antibiotics, and 14.6% (100/683) were prescribed both topical and systemic antibiotics [32]. Additionally, in an Indian study on drug utilization in the management of common skin illnesses, 17% of the 207 cases diagnosed with skin conditions were prescribed antibiotics [33]. Nevertheless, the presentations of dermatological conditions are quite varied, ranging from mere allergic irritations, fungal infections, viral and bacterial infections, and other injuries and secondary skin-related infections [34]. Ascertaining an aetiology for dermatological conditions can thus be tricky, making it difficult to tell whether there was a justified use of antibiotics in each of these cases [34]. It is, however, imperative that medical practitioners understand the need to use and prescribe antibiotics rationally for sustained efficacy.

Upper respiratory infection (URTI) was another empirically diagnosed condition in the current study, and antibiotic prescriptions among patients with URTI were almost double those with other disease conditions. This is comparatively higher than that in another study in Indonesia on the use of antibiotics in URTI, where 44% of the cases attended were prescribed antibiotics [35]; in another study involving antibiotic prescriptions for URTI in children under the age of five in China, 27.5% of all the 92,821 consultations of children between 3 to <5 years were prescribed antibiotics, accounting for over 60% of all the antibiotic prescriptions [36]. Additionally, in a study on antibiotic prescribing for URTI outpatients during influenza seasons involving 14,947 outpatients, over 40% were prescribed antibiotics, 41% of whom had conditions not requiring antibiotic therapy [37].

The limitation of this study is that the observations do not account for the accuracy of the written diagnosis and how patients’ history and physical examinations were recorded. However, the study gives a clue on the general practices of prescribers in low-level healthcare facility settings and provides information on which interventions can be made to improve their antibiotic prescribing skills in a more concerted effort against both the urgency and spread of antibiotic resistance.

## 5. Conclusions

In this study, we report high rates of antibiotic prescriptions and poor adherence to Standard Treatment Guidelines (STG) among prescribers in primary healthcare facilities. Prescribers with a diploma in clinical medicine were much more likely to prescribe antibiotics. There was an increase in the odds of antibiotic prescriptions among patients empirically diagnosed with urinary tract infections (UTIs), upper respiratory illness (URTI) and pneumonia.

## Figures and Tables

**Table 1:** Demographic information.

Variable	Frequency	Percentage
Age of patients		
<5	474	46.43
5–18	140	13.71
19–35	175	17.14
36–45	60	5.88
46–55	39	3.82
56+	133	13.03
District		
Chemba	390	38.20
Dodoma	631	61.80
Ownership		
Private	60	5.88
Government	961	94.12
Prescribers’ education		
Certificate	6	0.59
Diploma	966	94.61
Degree	49	4.80
Type of health facility		
Dispensary	748	73.26
Health centre	273	26.74
Sex		
Male	464	45.45
Female	557	54.55

**Table 2:** Association of antibiotics prescriptions by social, demographic and clinical predictors.

Variable	Non-Antibiotics (N = 242)	Antibiotics (N = 779)	X^2^	*p* Value
	N (%)	N (%)		
Age			7.1384	0.2105
<5	124 (26.16)	350 (73.84)		
5–18	38 (27.14)	102 (72.86)		
19–35	37 (21.14)	138 (78.86)		
36–45	10 (16.67)	50 (83.33)		
46–55	9 (23.08)	30 (76.92)		
56+	24 (18.05)	109 (81.95)		
District			1.6815	0.1947
Chemba	101 (25.90)	289 (74.10)		
Dodoma	141 (22.35)	490 (77.65)		
Ownership			0.7560	0.3846
Private	17 (28.33)	43 (71.67)		
Government	225 (23.41)	736 (76.59)		
Level of prescriber education			5.4522	0.0195
Degree	19 (37.25)	32 (62.75)		
Diploma	223 (22.99)	747 (77.01)		
Type of health facility			0.6126	0.4338
Dispensary	182 (24.33)	566 (75.67)		
Health centre	60 (21.98)	213 (78.02)		
Sex			4.2948	0.0382
Male	118 (21.18)	439 (78.82)		
Female	124 (26.72)	340 (73.28)		
Dermatological conditions			14.5453	0.0001
No	220 (22.59)	754 (77.41)		
Yes	22 (46.81)	25 (53.19)		
Diarrhoea			0.3927	0.5309
No	221 (23.46)	721 (76.54)		
Yes	21 (26.58)	58 (73.42)		
Malaria			16.8512	<.0001
No	215 (22.35)	747 (77.65)		
Yes	27 (45.76)	32 (54.24)		
Other diseases			6.4661	0.0110
No	134 (21.07)	502 (78.93)		
Yes	108 (28.05)	277 (71.95)		
Pneumonia			14.0314	0.0002
No	241 (24.85)	729 (75.15)		
Yes	1 (1.96)	50 (98.04)		
PID				0.0101
No	235 (23.29)	774 (76.71)		
Yes	7 (58.33)	5 (41.67)		
URTI			3.7990	0.0513
No	212 (24.85)	641 (75.15)		
Yes	30 (17.86)	138 (82.14)		
UTI			43.8463	<0.0001
No	242 (26.98)	655 (73.02)		
Yes	0 (0.00)	124 (100.00)		

**Table 3:** Binary logistic analysis for predictors of antibiotic prescriptions.

Variable	Univariate Analysis		Multivariate Analysis	
	cOR [95% CI]	*p* Value	aOR [95% CI]	*p* Value
District				
Chemba	Ref		Ref	
Dodoma	1.215 [0.905, 1.630]	0.1951	1.142 [0.832, 1.568]	0.4121
Sex				
Male	Ref		Ref	
Female	0.726 [0.552, 0.984]	0.0376	0.768 [0.566, 1.028]	0.0872
Prescriber education				
Degree	Ref		Ref	
Diploma	1.989 [1.106, 3.577]	0.0217	2.511 [1.343, 4.692]	0.0039
URTI				
No	Ref		Ref	
Yes	1.913 [1.354, 2.701]	0.0002	1.709 [1.129, 2.587]	0.0113
Pneumonia				
No	Ref		Ref	
Yes	16.678 [2.271, 120.270]	0.0055	15.918 [2.150, 117.973]	0.0056
Malaria				
No	Ref		Ref	
Yes	0.340 [0.200, 0.582]	<0.0001	0.293 [0.160, 0.504]	<.0001
Dermatological conditions				
No	Ref		Ref	
Yes	0.341 [0.193, 0.600]	0.0003	0.321 [0.184, 0.631]	0.0008
Other diseases				
No	Ref		Ref	
Yes	0.686 [0.511, 0.908]	0.0112	0.744 [0.510, 1.074]	0.1190

cOR = Crude odds ratios; aOR = Adjusted odds ratios; Ref = Reference variable.

## Data Availability

All data generated or analyzed during this study are available from the corresponding author upon reasonable request.
